# Factor XII mutations, estrogen-dependent inherited angioedema, and related conditions

**DOI:** 10.1186/1710-1492-6-16

**Published:** 2010-07-28

**Authors:** Karen E Binkley

**Affiliations:** 1 Division of Clinical Immunology and Allergy, Department of Medicine, University of Toronto, Toronto, Ontario, Canada

## Abstract

The clinical, biochemical and genetic features of the conditions known as estrogen-dependent inherited angioedema, estrogen-associated angioedema, hereditary angioedema with normal C-1 inhibitor, type III angioedema, or factor XII angioedema are reviewed. Discussion emphasizes pathogenesis, diagnosis, and management.

## Review

Estrogen-dependent and estrogen-associated inherited angioedemas were first described in 2000 [1,2], and cases are increasingly recognized around the world [3-7]. Recent studies offer new insights into the pathogenesis and treatment of this condition, which have relevance not only to these patients, but to those with classic forms of hereditary angioedema as well. Encouraging information on treatment of estrogen-related angioedemas is becoming available.

## Classic forms of hereditary angioedema

Classic forms of clinically recognized hereditary angioedema (HAE), types I and II, are genetically heterogeneous autosomal-dominant disorders, characterized by decreased levels, or function, respectively, of the inhibitor for the first component of the complement pathway (C1-INH) (Online Mendelian inheritance of man [OMIM] 106100; http://www.ncbi.nlm.nih.gov/omim/) Characteristic nonerythematous, non-pruritic swelling of parts of the face, upper respiratory tract, gastrointestinal tract, genitalia, hands and/or feet occur due to increased production of bradykinin, formed as insufficient C1 INH activity fails to restrict the action of factor XII and kallikrein [8-10].

## Estrogen related angioedemas: nomenclature, clinical and biochemical features

Novel forms of inherited angioedema, either completely dependent on, or associated with high estrogen levels, but otherwise clinically indistinguishable from classic forms of HAE, were independently reported by North American and European investigators in 2000 [1,2]. Cases are increasingly recognized around the world [3-7]. The nomenclature of these conditions is evolving as their underlying genetic abnormalities are elucidated. Originally referred to by clinical phenotype as estrogen-dependent (or estrogen-associated) inherited angioedema (EDIA, EAIA) [1], HAE with normal C1-INH activity [2]; or simply distinguished from classic forms as HAE type III [OMIM 610618] [2], the terms Factor XII-HAE or HAE-FXII have been used to identify the condition when associated with the recently identified gain-of-function mutation in the gene encoding factor XII (*F 12*) [11,12]. Some clinically indistinguishable cases do not carry this mutation [11], so underlying genetic diversity is apparent, and the nomenclature to describe these conditions will likely continue to evolve.

Clinical heterogeneity is evident in described cases. In a large multigenerational family of Italian origin, affected individuals experienced angioedema only during pregnancy, use of oral contraceptives or hormone replacement therapy [1]. In contrast, in different European families, phenotypes were far more variable [2]. Some patients experienced angioedema prior to menarche, with exacerbations after puberty and/or with high estrogen states, but in many cases, angioedema occurred even in low or normal estrogen level states. Initial reports [1,2] described only affected female patients, with an unaffected obligate male carrier [1]. More recently, pedigrees with affected male members have been described [13-15].

In one of the original reports [1], ethical considerations precluded the study of biochemical features during symptomatic episodes, as the index patients presented in the postmenopausal period, and none of their daughters became pregnant during the period of observation. As multiple family members had experienced laryngeal edema during high estrogen states, investigators reasoned that administration of estrogen could have life-threatening consequences, and affected individuals and individuals of unknown phenotype were advised to avoid estrogen. Indeed, death due to sudden airway obstruction was reported in some family members in the other originally reported pedigrees [2]. Thus, the only available biochemical analyses, performed when the affected individuals were asymptomatic, including normal C1-INH quantitative and functional assays, C3, C4,, and factor XII levels, at the time did not allow the investigators to preclude abnormalities in these parameters during symptomatic periods [1]. In the other initial report [2], biochemical analyses were reported in some symptomatic patients. C1 inhibitor level and activity, C3 and C4 were normal, even during acute attacks. These observations helped to distinguish EDIA and EAIA as being pathogenetically distinct from classic forms of HAE.

## Genetic features

The mode of inheritance could not be determined precisely in either of the original reports. Autosomal dominant transmission was considered most likely in the pedigree with strict estrogen dependence, though other modes of transmission could not be excluded [1,2]. Detailed information was reported in two multigenerational European pedigrees [2], one of which showed transmission of disease to children from an unaffected female, a phenomenon not seen in other reported pedigrees. Investigators speculated that the restriction to women suggested an X-linked dominant mode of inheritance; autosomal dominant transmission with hormonal control of the expression of the trait (the favoured explanation for the pedigree in the strictly estrogen-dependent pedigree) was thought to be less likely due to onset of symptoms in childhood, prior to significant hormonal effects. Autosomal dominant transmission seemed likely in a French pedigree [3] The recent identification heterozygosity for a gain-of-function mutation in *F12 *in female subjects in patients with EAIA [5,11,12,15,16] and EDIA, including those from the originally reported pedigree of Italian origin [17] suggests that autosomal dominant transmission is likely. However, the involvement of other genetic polymorphisms likely contributes to the diversity of clinical phenotypes [17].

In the family of Italian origin, the coding sequences as well as the 5' untranslated region (UTR) of the gene encoding C1 INH (*SERPING1*) were determined to be normal, clearly establishing this condition as being separate from the classic forms of hereditary angioedema (characterized by mutational inactivation of the C1 inhibitor gene). The 5' UTR of *F12 *(known to contain an estrogen-response element, alteration of which might explain the clinical phenotype of estrogen dependence) was also determined to be normal [1].

The biochemical and genetic observations from these two studies indicated that abnormalities in C1 INH could be excluded, and efforts to find the underlying cause of EDIA/EAIA were redirected elsewhere.

On the basis of co-segregation patterns, two different missense mutations in 6 index patients of 20 families (confirmed in 22 additional family members), mapping to 5 q 33-qter of F12 (Online Mendelian Inheritance in Man, [OMIM] 610619) were identified in European pedigrees of hereditary angioedema with normal C1-INH. Both in exon 9, one involved a threonine-to-lysine substitution (Thr309 Lys); the other a threonine-to arginine substitution (Thr309Arg) [11]. The presence of Thr328Lys in the family of Italian origin with estrogen-dependent angioedema was confirmed in affected family members living in Canada [17] and in Italy (R. Colombo, personal communication),

In addition, affected family members living in Canada were found to have polymorphisms in the genes for angiotensin-converting enzyme (ACE) and aminopeptidase P (APP) that are associated with lower circulating levels of these enzymes that are responsible for the degradation of bradykinin and its active metabolite [17]. Insertion/deletion polymorphisms in the ACE gene (*ACE*) account for 50% of the variability in human serum levels of ACE [18], with the insertion (I) allele associated with lower expression of ACE mRNA, and decreased degradation of bradykinin [19]. All three index patients had at least one copy of the inserted allele (I) in intron 16 of the ACE gene that is associated with lower levels of ACE.

Genetic variants in the gene encoding APP (*XPNPEP2*), resulting in reduced enzyme activity, higher bradykinin and des-Arg9-BK have been associated with angioedema induced by ACE inhibitors [20]. All three affected female subjects also had at least one copy of the A allele at the SNP rs3788853 locus, located 5' of *XPNPEP2*, which codes for membrane-bound APP, and is associated with decreased APP activity, decreased bradykinin and des-Arg9-BK degradation, and angioedema induced by ACE inhibitors [20,21]

Additional families with HAE and normal C1 inhibitor have been identified as carrying the Thr328Lys mutation [5,12,15,16,22], while other factor XII mutations have been described in different pedigrees [23].

## Bradykinin accumulation: the final common pathway

A new picture is emerging of the hereditary angioedemas as a group of genetically heterogeneous disorders of bradykinin metabolism, leading to its periodic accumulation. Bradykinin and its active metabolite, des-Arg9-BK, are the key mediators of angioedema [9,10,24,25]. Not only can mutations in different components (C1 INH, factor XII, ACE, APP, and as yet other, unidentified, factors) of bradykinin-related pathways occur, multiple different mutations can occur in each factor, and it seems likely that different combinations of these mutations contribute to the observed clinical heterogeneity of the conditions. In addition, the unique sensitivity of many of these components in bradykinin-related pathways to androgens and estrogens further modifies the clinical presentations. An appreciation of the pathways that result in the formation and degradation of bradykinin, and its active metabolite, des-Arg9-BK, and their regulation by sex hormones, contributes to the rational treatment of both classical and estrogen-dependent/factor XII- associated forms of hereditary angioedema.

## Effects of sex hormones on bradykinin pathways, and contribution to clinical phenotype

Before considering the influence of the sex hormones on key enzymes if bradykinin pathways, outlined below, it is helpful to review key aspects of the reciprocal regulation of bioavailable estrogen and testosterone through their effects sex hormones binding globulin (SHBG) (reviewed in [26]).

The activity of estrogen and testosterone is determined by the free or bioavailable fraction. In males, approximately 65% of testosterone circulates bound to SHBG, 78% in females. This bound fraction is essentially a reservoir; only the remaining free testosterone is biologically active. The fraction of estrogen bound to SHBG is less; only 30% is bound in males, 58% in females. The clinical relevance of this differential binding is apparent as abnormal variants of SHBG that bind sex hormones less efficiently result in a preferential increase in bioavailable testosterone with resulting masculinization.

By influencing the level of SHBG, each of the sex hormones enhances its own bioavailability, while decreasing the relative bioavailability of the other. For example, estrogen increases levels SHBG, this in turn binds more testosterone than estrogen, increasing the relative bioavailability of estrogen. Conversely, androgens decrease levels of SHBG, resulting in a preferential increase in the bioavailability of androgens. This type of negative reciprocal regulation of bioavailability can amplify the effects of small changes in the relative amounts of estrogen versus testosterone, and may in part explain the exquisite sensitivity of the clinical phenotype to relatively small changes in sex hormones levels. Danazol has been shown to suppress SHBG levels in classic HAE patients [27], although other observations suggest there may be additional effects of SHBG [28].

### Estrogen: effect on bradykinin production

#### Factor XII

High levels of estrogen, such as occurr during pregnancy or oral contraceptive use [29,30], are associated with increased levels of factor XII, likely due to an estrogen-response element in the promoter region of the gene [31,32]. When activated, factor XII converts pre-kallikrein to kallikrein, which produces bradykinin from high molecular weight kininogen. Under the conditions of high estrogen levels, the increased availability of factor XII for activation would favor increased bradykinin production.

#### C-1 INH

High levels in estrogen during pregnancy [33-35], or oral contraceptive use [36],are associated with reduced levels of C-1 INH. As C-1 INH normally inhibits activated factor XII and kallikrein; reduced inhibition of factor XII and kallikrein with high estrogen levels would favour increased bradykinin production.

### Estrogen: effect on bradykinin degradation

#### ACE

Estrogen suppresses ACE expression [37]. As ACE is important both for the degradation of bradykinin and its active metabolite, des-Arg9-BK, reduced levels of ACE under conditions of high estrogen levels result in reduced degradation of bradykinin and its active metabolite, favouring their accumulation.

#### APP

The effect of estrogen on APP levels is not known. However, it has been reported that androgens increase APP levels [38], and, as estrogen increases SHBG, and reduces bioavailability of testosterone, it is reasonable to speculate *that *estrogen might reduce APP levels. As APP is particularly important in the degradation ofdes-Arg9-BK, and to a lesser extent bradykinin itself, reduced APP levels would favor the accumulation of bradykinin.

### Androgens: effect on bradykinin production

#### C-1 INH

Androgens increase the level of C-1 INH [39,40], which in turn inhibits activated factor XII and kallikrein, reducing bradykinin formation.

#### Factor XII

In rats, danazol was found to increase factor XII [41]. Specific studies in humans could not be located. Given the clinical efficacy of attenuated androgens in classic HAE, one might speculate that the clinically beneficial effects on other components of the bradykinin pathway (increased C-1 INH, increased APP, with secondary effects of the relative bioavailability of estrogen) outweigh the effect of increased factor XII. However, this observation has intriguing consequences for HAE-FXII. In this situation, androgen-induced increase in the overactiveThr328Lys factor XII could be deleterious. This has not been observed clinically [16], suggesting that, as in classic HAE, beneficial effects of androgens on other components of bradykinin metabolism overweight their effects on factor XII.

### Androgens: effect on bradykinin degradation

#### APP

Androgens increase APP levels [38]which would favor bradykinin degradation.

#### ACE

Animal studies suggest androgens are responsible for increased ACE levels [42,43]. Studies specifically addressing the influence of androgens on human ACE levels could not be located.

In summary, androgens and estrogens have reciprocal, antagonistic effects on bradykinin metabolism through their effects on multiple components in these pathways relevant to the pathogenesis and treatment of classic and estrogen-related HAEs. Primary effects result in direct modification of the levels of key components in the pathways for bradykinin formation and degradation. Secondary effects, mediated through alterations in the level of SBHG, may amplify these primary effects by changing the relative bioavailability of the opposing sex hormone. High estrogen levels result in conditions favorable to increased bradykinin accumulation, whereas high androgen levels result in conditions that lead to low levels of bradykinin. The reciprocal antagonistic effects on multiple key components of bradykinin metabolism likely account for the sensitivity of disease expression to small changes in hormone levels. Exquisite sensitivity is most evident in patients with a strict estrogen-dependent phenotype [1]. For example, affected members in the family with the *F 12 *Thr328Lys mutation, the I allele of *ACE*, and the A allele of rs3788853 at the *XPNPEP2 *locus of the APP gene never experienced angioedema during normal menstrual cycles; however, angioedema occurred during pregnancy within days of the first missed menstrual period, a time when estrogen levels would be only marginally higher than the end of a normal cycle.

## Diagnosis

The diagnosis of estrogen-related HAEs remains challenging as there is no specific, readily available assay. They should be suspected in the setting of otherwise unexplained episodes of angioedema, occurring in, or made worse, by high estrogen states, noting that strict estrogen dependence does not occur in every pedigree, even those with established factor XII Thr328Lys mutations [16]. Classic forms of HAE can also be exacerbated by high estrogen states, but these can be excluded if C-4, C-1 INH function and C-1 INH activity are normal [44]. Genetic analysis of suspected cases has been performed a research basis, however, the methodology required is likely within the capabilities of tertiary genetic referral centres. Identification of pre-symptomatic individuals in established pedigrees should be a priority so that exogenous estrogens (primarily oral contraceptives in young women) and the possibility of laryngeal edema can be avoided.

## Treatment: avoidance of aggravating medications

Two distinct classes of medications contribute to bradykinin accumulation and should be avoided. Exogenous estrogens (oral contraceptives and hormone replacement therapy) have multiple effects that favour bradykinin accumulation, and have been associated with clinical exacerbations in both the estrogen-related [16] and classic forms of HAE [44]. Cardiovascular medications, ACE inhibitors, act at single point in bradykinin degradation. They have been associated with exacerbation of angioedema in both classic and estrogen-related HAEs. One patient experienced worsening of HAE-FXII with the angiotensin II receptor blocker losartan [16]; the mechanism for this effect is unclear. It would seem prudent to avoid angiotensin receptor blockers in patients with estrogen-associated HAE, if possible.

## Treatment: acute management

Treatment experience of this newly recognized condition is limited; there are no well controlled trials. C1-INH concentrate was moderately or very effective in 6/7 patients experiencing 63 angioedema attacks [16]. Presumably, the additional C-1 INH achieved this clinical outcome by inhibiting activated factor XII and kallikrein, preventing the positive feedback loops that amplify their activity. The risks associated with this treatment would be those associated with the use of blood products. It is unclear if any of these reported uses occurred during pregnancy. Recombinant C-1 INH would be expected to have similar effects, but the potential for blood-borne infections would be eliminated.

Fresh frozen plasma (FFP), is effective in classic forms of HAE [45]; its use is considered if C-1 INH concentrates are not readily available to treat an acute attack. Consideration of mechanisms responsible for bradykinin accumulation in estrogen-related angioedemas suggests FFP might be useful in these conditions. With respect to factor XII, transfusion of FFP (with normal factor XII activity) might be expected to dilute theThr328Lys factor XII with increased activity, helping to return overall factor XII activity towards normal, thereby reducing further bradykinin formation. With respect to C1-INH, transfusion of FFP would help replace any C-1 INH consumed by uncontrolled factor XII and kallikrein activation, helping to restore appropriate levels of inhibition of factor XII and kallikrein. With respect to the enzymes responsible bradykinin degradation, ACE and APP, transfusion of FFP would supplement levels in individuals having low levels of these enzymes due to genetic polymorphisms of their corresponding genes, as in individuals described [17]. Therefore, there is a theoretical basis for the use of FFP in estrogen-related angioedemas if C-1 INH concentrates are not readily available to treat an acute attack.

Ecallantide, is a potent, selective, reversible inhibitor of kallikrein [46] that has recently become available for clinical use. This compound blocks the binding site of kallikrein, and reduces the conversion of high molecular weight kininogen (HMWK) to bradykinin. It also prevents the positive feedback loop in which kallikrein increases activation of factor XII, enhancing further kallikrein production. This compound has been shown to be effective in treating acute episodes of angioedema in classic HAE [47]. There are no published reports of its use in the estrogen-related angioedemas. No published data regarding use in pregnancy could be located.

Icatibant, a bradykinin receptor-2 antagonist has been shown to be effective in ameliorating acute attacks of classic HAE [48]. It may be useful in the estrogen-related angioedemas [49].Safety during pregnancy is not established.

Ineffective treatments include corticosteroids, in 27 patients, and antihistamines in 15 patients, which were ineffective in controlling acute attacks [16], as is seen in patients with classic HAE.

## Treatment: prophylaxis

Progesterone use has been reported. Eight women on various progesterone-only preparations were symptom free during progesterone treatment [16], but the frequency of previous attacks and whether these occurred only during high estrogen states is not reported, so it is difficult to evaluate whether the absence of symptoms was attributable to the use of progesterone, or the avoidance of estrogen. Further studies of the efficacy of progesterone seem warranted in patients who experience ongoing symptoms despite estrogen avoidance. However, caution is warranted as high progesterone levels have been associated with a higher number of episodes of angioedema in classic HAE [28].

Danazol use has been reported. Two patients experienced amelioration of symptoms with danazol [16]. Though not stated specifically, it seems likely that symptoms occurred during normal estrogen states. Attenuated androgens act at many points in bradykinin pathways to favour lower levels of bradykinin, thereby ameliorating symptoms. Androgens have been a cornerstone of treatment of classic HAEs for decades. However, they are contraindicated in pregnancy due to their masculinization of the fetus. The use of androgens would likely be limited to patients who experience ongoing symptoms despite estrogen avoidance, i.e., cases without strict estrogen dependence. For example, in the family with the strict EDIA phenotype [1] women of childbearing age were asymptomatic if they avoided oral contraceptives and used alternate methods of birth control, so androgens were not required. Postmenopausal individuals were asymptomatic if they avoided hormone replacement therapy (one affected individual with severe menopausal symptoms was successfully managed with very low dose transdermal estrogen without recurrence of angioedema, K. Binkley, unpublished observation), so androgens were not required. In this pedigree, identification of the phenotype allowed symptoms to be successfully managed by avoidance of triggers. Pregnancy was the only state during which treatment would be required, when androgens are contraindicated.

Tranexamic acid is used in classic forms of HAE, but its efficacy is generally lower than that of the attenuated androgens. It is thought that this antifibrinolytic agent acts through the inhibition of plasmin. There is risk of thromboembolic events with its use. Tranexamic acid was used successfully in one patient with estrogen-related angioedema [16]. It would seem the primary use of this agent would be in cases in which angioedema continued despite avoidance of estrogens.

In summary, various treatment options are available for patients with estrogen-related angioedema that is not controlled despite avoidance of exogenous estrogens, though data is limited. The greatest need is for safe and effective treatments for patients who desire pregnancy. Currently, C-1 INH replacement with concentrates or recombinant C-1 INH seemed to be the best options.

## Conclusions

In the decade since their original description, significant progress has been made in characterizing the underlying responsible genetic abnormalities in the estrogen-related HAEs. Significant clinical and genetic heterogeneity in these conditions is apparent, and it is likely that multiple genetic factors contribute to disease expression, even within the same pedigree. By extension, some of the more common genetic polymorphisms contributing to increased bradykinin accumulation, reported in patients with EDIA, might also contribute to the well-recognized phenotypic heterogeneity within individual pedigrees of classic HAEs. The emerging picture is that both classic and estrogen-related HAEs belong to a family of diverse genetic disorders of bradykinin metabolism that favour its periodic accumulation, resulting in angioedema. In both classic and estrogen-related HAEs, the profound effects of estrogens and androgens on multiple components in bradykinin metabolism pathways contribute to the expression of clinical phenotype, and have important implications for treatment. Limited data are encouraging that C-1 INH replacement is effective in treating acute attacks caused by mutations in *F 12*. Ecallantide and icatibant may also be useful, but further studies will be required. Optimal management of estrogen-related angioedemas remains to be determined. Currently, definitive diagnosis remains challenging as genetic analysis is not immediately available to most clinicians. As these conditions are increasingly recognized, and the need for access to this analysis becomes apparent, specialized tertiary and quaternary genetic centres may be able to offer analysis in carefully selected patients. The most pressing needs relate to treatment during pregnancy, the one high-estrogen state that patients may be unwilling to avoid, and the one in which agents for long-term prophylaxis (androgens and tranexamic acid) are contraindicated, and safety data on agents used to treat acute attacks (C-1 INH replacement, kallikrein inhibitors, and bradykinin receptor antagonists) is almost nonexistent. Large controlled trials of treatment will be challenging due to the heterogeneity and rarity of these conditions.

## Abbreviations

ACE: angiotensin converting enzyme; APP: aminopeptidase P; C-1 INH: inhibitor of the first component of *the *complement pathway; DES- ARG9-BK: des- Arginine9 bradykinin; EAIA: estrogen-associated inherited angioedema; EDIA: estrogen-dependent inherited angioedema; *F12*: gene encoding *factor *XII; HAE: hereditary angioedema; I/D: insertion/deletion; UTR: untranslated region; *XPNEPEP2*: gene encoding aminopeptidase P;

## Competing interests

The author declares that they have no competing interests.

## Funding

Publication costs were supplied through an unrestricted grant from the Canadian Hereditary Angioedema Network (CHAEN)/Réseau Canadien d'angioédème héréditaire (RCAH)

## References

[B1] Binkley KE Davis A Clinical, biochemical, and genetic characterization of a novel estrogen-dependent inherited form of angioedema J Allergy Clin Immunol 2000 106 546 50 10.1067/mai.2000.108106 10984376

[B2] Bork K Barnstedt SE Koch P Traupe H Hereditary angioedema with normal C1-inhibitor activity in women Lancet 2000 356 213 7 10.1016/S0140-6736(00)02483-1 10963200

[B3] Martin L Degenene D Toutain A Ponard D Watier H Hereditary angioedema type III: an additional French pedigree with autosomal dominant transmission J Allergy Clin Immunol 2001 107 747 8 10.1067/mai.2001.114242 11295676

[B4] Herrmann G Schneider L Krieg T Hunzelmann N Scharfetter-Kochanek K Efficacy of danazol treatment in a patient with a new variant of hereditary angiooedema (HAE III) Br J Dermatol 2004 150 157 8 10.1111/j.1365-2133.2004.05669.x 14746637

[B5] Bouillet L Ponard D Rousset H Cichon S Drouet C A case of hereditary angioedema type III presenting with C1-inhibitor cleavage and a missense mutation in the F12 gene Br J Dermatol 2007 156 1063 5 10.1111/j.1365-2133.2007.07778.x 17381464

[B6] Fiz Matias J Ferrer Ceron SM Garcia Perez C Marcos Vidal JM Analgesia obstetrica en un caso de edema angioneurotico hereditario tipo III Rev Esp Anestesiol Reanim 2007 54 253 4 17518179

[B7] Serrano C Guilarte M Tella R Dalmau G Bartra J Gaig P Cerda M Cardona V Valero A Oestrogen-dependent hereditary angiooedema with normal C1 inhibitor: description of six new cases and review of pathogenic mechanisms and treatment Allergy 2008 63 735 41 10.1111/j.1398-9995.2007.01579.x 18070231

[B8] Cyr M Eastlund T Blais C Jr Rouleau JL Adam A Bradykinin metabolism and hypotensive transfusion reactions Transfusion 2001 41 136 50 10.1046/j.1537-2995.2001.41010136.x 11161259

[B9] Davis AE The pathogenesis of hereditary angioedema Transfus Apheresis Sci 2003 29 195 203 10.1016/j.transci.2003.08.012 14572810

[B10] Davis AE C1 inhibitor and hereditary angioneurotic edema Ann Rev Immunol 1988 6 595 628 10.1146/annurev.iy.06.040188.003115 3289579

[B11] Dewald G Bork K Missense mutations in the coagulation factor XII (Hageman factor) gene in hereditary angioedema with normal C1 inhibitor Biochem Biophys Res Commun 2006 343 1286 9 10.1016/j.bbrc.2006.03.092 16638441

[B12] Cichon S Martin L Hennies HC Muller F Van Driessche K Karpushova A Stevens W Colombo R Renne T Drouet C Bork K Nothen MM Increased activity of coagulation factor XII (Hageman factor) causes hereditary angioedema type III Am J Hum Genet 2006 79 1098 104 17186468 10.1086/509899 PMC1698720

[B13] Bork K Gul D Dewald G Hereditary angioedema with normal C1 inhibitor in a family with affected women and men Br J Dermatol 2006 154 542 5 10.1111/j.1365-2133.2005.07048.x 16445789

[B14] Bork K Gul D Hardt J Dewald G Hereditary angioedema with normal C1 inhibitor: clinical symptoms and course Am J Med 2007 120 987 92 10.1016/j.amjmed.2007.08.021 17976427

[B15] Martin L Raison-Peyron N Nothen MM Cichon S Drouet C Hereditary Angioedema with normal C1 inhibitor gene in a family with affected women and men is associated with the p.Thr328Lys mutation in the F12 gene J Allergy Clin Immunol 2007 120 975 7 10.1016/j.jaci.2007.07.002 17825897

[B16] Bork K Wulff K Hardt J Witzke G Staubach P Hereditary angioedema caused by missense mutations in the factor XII gene: clinical features, trigger factors, and therapy J Allergy Clin Immunol 2009 124 129 34 10.1016/j.jaci.2009.03.038 19477491

[B17] Duan QL Binkley K Rouleau GA Genetic analysis of factor XII and bradykinin catabolic enzymes in a family with estrogen-dependent inherited angioedema J Allergy Clin Immunol 2009 123 906 10 10.1016/j.jaci.2008.12.010 19178938

[B18] Rigat B Hubert C Alhenc-Gelas F Cambien F Corvol P Soubrier F An insertion/deletion polymorphism in the angiotensin I-converting enzyme gene accounting for half the variance of serum enzyme levels J Clin Invest 1990 86 1343 6 1976655 10.1172/JCI114844 PMC296868

[B19] Murphey LJ Hachey DL Oates JA Morrow JD Brown NJ Metabolism of bradykininin vivo in humans: identification of BK1-5 as a stable plasma peptide metabolite J Pharmacol Exp Ther 2000 294 263 9 10871321

[B20] Duan QL Nikpoor B Dube MP Molinaro G Meijer IA Dion P Rochfort D Saint- Onge J Flury L Brown NJ Gainer JV Rouleau JL Agostini A Cugno M Simon P Clavel P Potier J Webhe B Bernarbis S Marc-Aurele J Chanard J Foroud T Adam A Rouleau GA A variant inXPNPEP2 is associated with angioedema induced by angiotensin I-converting enzyme inhibitors Am J Hum Genet 2005 77 617 26 16175507 10.1086/496899 PMC1275610

[B21] Molinaro G Duan QL Chagnon M Moreau ME Simon P Clavel P Lavaud S Boileau G Rouleau GA Lepage Y Adam A Chanard J Kinin dependent hypersensitivity reactions in hemodialysis: metabolic and genetic factors Kidney Int 2006 70 1823 31 10.1038/sj.ki.5001873 17003818

[B22] Bell CG Kwan E Nolan RC Baumgart KW First molecular confirmation of an Australian case of type III hereditary angioedema Pathology 2008 40 82 3 10.1080/00313020701716433 18038321

[B23] Hentges F Hilger C Kohnen M Gilson G Angioedema and estrogen-dependent angioedema with activation of the contact system J Allergy Immunol Clin 2009 123 262 4 10.1016/j.jaci.2008.10.056 19130939

[B24] Blais C Jr Rouleau JL Brown NJ Lepage Y Spence D Munoz C Friborg J Geadah D Gervais N Adam A Serum metabolism of bradykinin and des-Arg9-bradykinin in patients with angiotensin-converting enzyme inhibitor-associated angioedema Immunopharmacology 1999 43 2-3 293 302 10.1016/S0162-3109(99)00133-2 10596866

[B25] Molinaro G Cugno M Perez M Lepage Y Gervais N Agostoni A Adam A Angiotensin-converting enzyme inhibitor-associated angioedema is characterized by a slower degradation of des-arginine(9)-bradykinin J Pharmacol Exp Ther 2002 303 1 232 7 10.1124/jpet.102.038067 12235256

[B26] Strauss JF Barbieri R editors Yen and Yaffe's Reproductive Endocrinology 2009 Chapter 4 Sixth Saunders Elselvier 79 104

[B27] Schwarz S Tappeiner G Hintner H Hormone binding globulin levels in patients with hereditary angiooedema during treatment with Danazol Clin Endocrinol (Oxf) 1981 14 6 563 70 10.1111/j.1365-2265.1981.tb02966.x 6794961

[B28] Visy B Fust G Varga L Szendei G Takacs E Karadi I Fekete B Harmat G Farkas H Sex hormones in hereditary angioneurotic oedema Clin Endocrinol 2004 60 4 508 15 10.1111/j.1365-2265.2004.02009.x 15049967

[B29] Gordon EM Ratnoff OD Saito H Donaldson VH Pensky J Jones PK Rapid fibrinolysis, augmented Hageman factor (factor XII) titres and decreased C1 esterase inhibitor titres in women taking oral contraceptives J Lab Clin Med 1980 96 762 9 7419960

[B30] Gordon EM Williams SR Frencheck B Mazur CA Speroff I Dose dependent effects of postmenopausal estrogen and progestin on antithrombin III and factor XII J Lab Clin Med 1988 111 52 6 3335825

[B31] Klein-Hitpass L Tsai SY Greene GL Clark JH Tsai MJ O'Malley BW Specific binding of estrogen receptor to the estrogen response element Mol Cell Biol 1989 9 43 9 2927397 10.1128/mcb.9.1.43-49.1989 PMC362143

[B32] Farsetti A Misiti S Citarella F Felici A Andreoli M Fantoni A Sacchi A Pontecorvi A Molecular basis of estrogen regulation of Hageman factor XII gene expression Endocrinology 1995 136 5076 83 10.1210/en.136.11.5076 7588244

[B33] Ogston D Walker J Campbell DM C1 inactivator level in pregnancy Thromb Res 1981 23 454 5 10.1016/0049-3848(81)90206-1 7324006

[B34] Halbmayer WM Hopmeier P Mannhalter C Heuss F Leodolter S Rubi K Fischer M C1 esterase inhibitor in uncomplicated pregnancy and mild and moderate preeclampsia Thromb Haemost 1991 2 134 8 2053098

[B35] Cohen AJ Laskin C Tarlo S C1 esterase inhibitor in pregnancy J AllergyClin Immunol 1992 90 412 3 10.1016/S0091-6749(05)80025-9 1527327

[B36] Jespersen J Kluft C Increased englobulin fibrinolytic potential in women on oral contraceptives low in oestrogen levels of extrinsic and intrinsic plasminogen activators, prekallikrein, factor XII and C1 inactivator Thromb Haemost 1985 54 454 9 2417350

[B37] Stevenson JC Olatdipo A Manassiev N Whitehead MI Guilford S Proudler AJ Randomized trial of effect of transdermal cutaneous combined hormone replacement therapy on cardiovascular risk markers B J Haematol 2004 124 802 8 10.1111/j.1365-2141.2004.04846.x 15009069

[B38] Drouet C Desormeaux A Robillard J Ponard D Bouillet L Martin L Kanny G Moneret-Vautrin DA Bosson JL Quesada JL López-Trascasa M Metallopeptidase activities in hereditary angioedema: effect androgen prophylaxis on plasma aminopeptidase P J Allergy Clin Immunol 2008 121 429 33 10.1016/j.jaci.2007.10.048 18158172 PMC4126900

[B39] Gelfand JA Sherins RJ Alling DW Frank MM Treatment of hereditary angioedema with danazol. Reversal of clinical and biochemical abnormalities N Engl J Med 1976 295 1444 8 10.1056/NEJM197612232952602 792688

[B40] Cicardi M Zingale L Clinical manifestations of hereditary angioedema J Allergy Clin Immunol 2004 114 Suppl S55 8 10.1016/j.jaci.2004.06.021 15356570

[B41] Nobukata H Katsuki Y Ishikawa T Inokuma M Shibutani Y Effect of dienogest on bleeding time, coagulation, fibrinolysis, and platelet aggregation in female rats Toxicol Lett 1999 104 1-2 93 101 10.1016/S0378-4274(98)00354-3 10048754

[B42] Freshour JR Chase SE Vikstrom KL Gender differences in cardiac ACE expression are normalized in androgen-deprived male mice Am J Physiol Heart Circ Physiol 2002 283 5 H1997 2003 12384478 10.1152/ajpheart.01054.2001

[B43] Lim YK Retnam L Bhaqavath B Sethi SK bin Ali A Lim SK Gonadal effects on plasma ACE activity in mice Atherosclerosis 2002 160 2 311 6 10.1016/S0021-9150(01)00576-7 11849653

[B44] Bowen T Cicardi M Bork K Zuraw B Frank M Ritchie B Farkas H Varga L Zingale LC Binkley K Wagner E Adomaitis P Brosz K Burnham J Warrington R Kalicinsky C Mace S McCusker C Schellenberg R Celeste L Hebert J Valentine K Poon MC Serushago B Neurath D Yang W Lacuesta G Issekutz A Hamed A Kamra P Dean J Kanani A Stark D Rivard GE Leith E Tsai E Waserman S Keith PK Page D Marchesin S Longhurst HJ Kreuz W Rusicke E Martinez-Saguer I Aygören-Pürsün E Harmat G Füst G Li H Bouillet L Caballero T Moldovan D Späth PJ Smith-Foltz S Nagy I Nielsen EW Bucher C Nordenfelt P Xiang ZY Hereditary angiodema: a current state-of-the-art review, VII: Canadian Hungarian 2007 International Consensus Algorithm for the Diagnosis, Therapy, and Management of Hereditary Angioedema Ann Allergy Asthma Immunol 2008 100 1 Suppl 2 S30 40 10.1016/S1081-1206(10)60584-4 18220150

[B45] Pickering RJ Good RA Kelly JR Gewurz H Replacement therapy in hereditary angioedema. Successful treatment of two patients with fresh frozen plasma Lancet 1969 1 326 30 10.1016/S0140-6736(69)91295-1 4179348

[B46] Ladner R Kent R Ley A Nixon A Sexton D Discovery of Ecallantide: A Potent and Selective Inhibitor of Plasma Kallikrein J Allergy Clin Immunol 2007 119 S1 S312 10.1016/j.jaci.2006.12.591

[B47] MacGinnitie AJ Pullman WE Horn PT Interim Results from Continuation-the Ongoing, Open-Label, Extension Study of Ecallantide for the Treatment of Acute Attacks of Hereditary Angioedema J Allergy Clin Immunol 2010 2 S1 AB165

[B48] Bork K Frank J Grundt B Schlattmann P Nussberger J Kreuz W Treatment of acute edema at this is hereditary angioedema with a bradykinin receptor-2 antagonist (Icatibant) J Allergy Clin Immunol 2007 119 1497 1503 10.1016/j.jaci.2007.02.012 17418383

[B49] Bouillet L Boccon-Gibod I Ponard D Drouet C Cesbron JY Dumestre-Perard C Monnier N Lunardi J Massot C Gompel A Bradykinin receptor 2 antagonist (icatibant) for hereditary angioedema type III attacks Ann Allergy Asthma Immunol 2009 103 448 10.1016/S1081-1206(10)60369-9 19927548

